# Identification and functional analysis of cytochrome P450 complement in *Streptomyces virginiae* IBL14

**DOI:** 10.1186/1471-2164-14-130

**Published:** 2013-02-27

**Authors:** Zhi-Zhen Li, Xiao-Fei Li, Wei Yang, Xiang Dong, Jie Yu, Shu-Liang Zhu, Man Li, Li Xie, Wang-Yu Tong

**Affiliations:** 1 Integrated Biotechnology Laboratory, Institute of Health Science, School of Life Science, Anhui University, 111 Jiulong Road, Hefei 230601, China; 2 Department of Chemical Engineering, McMaster University, Hamilton, Ontario, L8S 4L7, Canada

**Keywords:** Biotransformation, Cytochrome P450, Ferredoxin, Ferredoxin reductase, Gene sequencing, Secondary metabolism

## Abstract

**Background:**

As well known, both natural and synthetic steroidal compounds are powerful endocrine disrupting compounds (EDCs) which can cause reproductive toxicity and affect cellular development in mammals and thus are generally regarded as serious contributors to water pollution. *Streptomyces virginiae* IBL14 is an effective degradative strain for many steroidal compounds and can also catalyze the C25 hydroxylation of diosgenin, the first-ever biotransformation found on the F-ring of diosgenin.

**Results:**

To completely elucidate the hydroxylation function of cytochrome P450 genes (CYPs) found during biotransformation of steroids by *S. virginiae* IBL14, the whole genome sequencing of this strain was carried out via 454 Sequencing Systems. The analytical results of BLASTP showed that the strain IBL14 contains 33 CYPs, 7 ferredoxins and 3 ferredoxin reductases in its 8.0 Mb linear chromosome. CYPs from *S. virginiae* IBL14 are phylogenetically closed to those of *Streptomyces sp.* Mg1 and *Streptomyces sp.* C. One new subfamily was found as per the fact that the CYP *Svu*001 in *S. virginiae* IBL14 shares 66% identity only to that (ZP_05001937, protein identifer) from *Streptomyces sp.* Mg1. Further analysis showed that among all of the 33 CYPs in *S. virginiae* IBL14, three CYPs are clustered with ferredoxins, one with ferredoxin and ferredoxin reductase and three CYPs with ATP/GTP binding proteins, four CYPs arranged with transcriptional regulatory genes and one CYP located on the upstream of an ATP-binding protein and transcriptional regulators as well as four CYPs associated with other functional genes involved in secondary metabolism and degradation.

**Conclusions:**

These characteristics found in CYPs from *S. virginiae* IBL14 show that the EXXR motif in the K-helix is not absolutely conserved in CYP157 family and I-helix not absolutely essential for the CYP structure, too. Experimental results showed that both CYP Svh01 and CYP Svu022 are two hydroxylases, capable of bioconverting diosgenone into isonuatigenone and β-estradiol into estriol, respectively.

## Background

Cytochrome P450 (CYP) genes refer to such genes that encode a superfamily of iron-containing hemoproteins with a maximum absorption spectrum near 450 nm, often characterized by conserved Cys residue in hydrophobic pocket(s) [1]. Most of the ORFs of CYP have three distinct characteristics used often for their identification and analysis, i.e., the I-helix of putative CYPs (a highly conserved threonine involved in oxygen activation), the conserved EXXR motif located in the K-helix and the cytochrome P450 cysteine heme-iron ligand signature motif (GXXXCXG, there are exceptions) [2]. According to a widely-accepted taxonomy, CYPs within a family share more than 40% amino acid identity and members of subfamilies share more than 55% amino acid identity [3]. Occasionally, the decision to accept a sequence in a known family depends greatly on how it clusters on a tree, not so much on the absolute amino acid identity [4].

CYPs have been confirmed existing in all eukaryotic (human, animals, plants, fungi, etc.) and prokaryotic organisms (bacteria, archaea, and even in viruse) [5-8]. They often are monooxygenases involved in oxidation of a range of endogenous compounds, such as cholesterol, lipids and steroidal hormones, as well as xenobiotics such as drugs and toxic chemicals in environment [9-11]. CYPs catalyse diverse reactions, including C-H hydroxylation, epoxidation, hetero-atom oxidation, aromatic ring oxidation and dealkylation [11-13]. In the catalytic reaction process of P450 monooxygenase, one atom of O_2_ is inserted into substrate while the other is reduced to H_2_O. CYP genes responsible for secondary metabolism are often laid in antibiotic biosynthetic gene clusters to catalyze stereo- and region- specific reaction of substrates to related derivatives.

The biotransforming capabilities of bacterial CYPs have been widely elucidated. P450soy (CYP105D1) from *Streptomyces griseus* was involved in the degradation of a diverse array of complex agrochemicals and environmental pollutants [14]. CYP105C1 from *Actinomycete* spp. had the ability to transform benanomicin A into two derivatives, 10-hydroxybenanomicin A and 11-O-demethylbenanomicin [15]. The functions of related CYP107 family members have been reported. CYP107E from *Micromonospora griseorubida* was found to govern the hydroxylation and epoxidization in mycinamicin biosynthesis [16], P450 Terf (107 L) from *Streptomyces platensis* to catalyze hydroxylation of terfenadine [17] and hydroxylase PikC (107 L1) of *Streptomyces venezuelae* to convert narbomycin to picromycin [18]. CYP124 of *Mycobacterium tuberculosis* demonstrated omega-hydroxylase activity of relevant methyl-branched lipids [19]. YbdT (CYP152A) of *Bacillus subtilis* was involved in fatty acid beta-hydroxylation [20]. CYP154 of *Nocardia farcinica* IFM10152 had the functions of the O-dealkylation and ortho-hydroxylation of formononetin [21] and 154H1 from *Clostridium acetobutylicum* performed biocatalytic reactions with different aliphatic and aromatic substrates [22].

Genome sequencing is an effective way to predict and annotate all the possible CYPs genes in an organism. *Streptomyces coelicolor* A3 (2), a typical strain which is often used for the study of physiological function and antibiotic production, is the first *Streptomyces* species sequenced in 2001. Its linear chromosome is 8.7 Mb [23] which contains 7825 open reading frames (ORFs) with 18 putative CYPs [24]. *S. avermitilis*, known for producing the antiparasitic agent avermectin, contains 7600 ORFs with 33 putative CYPs in the 9 Mb chromosomes [25,26]. The genome of *Streptomyces peucetius* ATCC27592 with the size of 8.7 Mb contains 19 putative CYPs [27].

*S. virginiae* IBL14, isolated from activated sludge for treatment of waste from a steroidal drug factory, is an effective degradative strain of various steroidal compounds, including progesterone, isotestosterone, dihydrotestosterone, hydrocortisone, cholesterol and ostrone [28]. To comprehensively understand the function of CYPs of *S. virginiae* IBL14 in degradation and biotransformation of diosgenin, the whole genome sequencing of *S. virginiae* IBL14 isolated by our lab was carried out for the first time. Using *in silico* technology, we predict and annotate all of the putative CYPs of *S. virginiae* IBL14 and analyze these CYPs evolutionarily and functionally via comparison with those of other *Streptomyces* species. Furthermore, functions and characteristics of CYP genes *svh*01 and CYP *svu*022 in this strain are experimentally identified and analyzed.

## Results and discussion

### Genome sequencing and CYPs in *S. virginiae* IBL14

By *in silico* analysis of newly-sequenced *S. virginiae* IBL14 8.0 Mb genome, 8288 ORFs are identified and the total GC content exceeds 70%. The annotated results via Rpsblast display that there are a total of 33 putative CYPs in the genome of this strain IBL14, contributing to approximately 0.4% of all the coding sequences. The number of CYPs is identical to that in *S. avermitili* and almost two times as that in *S. coelicolor* A3(2) and *S. peucetius* ATCC27952 (18 and 19 CYPs, respectively). Such high level of CYP diversity suggests the high diversity of the secondary metabolism pathways in *S. virginiae* IBL14.

The 32 out of 33 putative CYPs of *S. virginiae* IBL14 belong to 13 previously-reported CYP families, i.e., 105 (5), 107 (11), 121 (1), 124 (1), 147 (1), 152 (1), 154 (1), 157 (2), 185 (1), 191 (3), 197 (4), 247(1) and another to an unknown family, as shown in Table 1. Among the all, the CYP121A (*Svu*018), CYP124 (*Svu*19), CYP147 (*Svu*020), CYP152 (*Svu*021), CYP154H (*Svu*022), CYP157 (*Svu*023-024), CYP185 (*Svu*025), CYP191 (*Svu*26-28), CYP197 (*Svu*017,029-031) and CYP247 (*Svu*032) are firstly reported in *S. virginiae*, and especially, CYP107M, CYP185A and CYP247A have been found rarely in *Streptomycete* spp. The *Svu*025, *Svu*026 and *Svu*029 have lower identity with other family members (<50%) while others show more than 63% identity to CYPs of other organisms. It’s worth noting that the *Svu*001 presumably belongs to a new CYP family since no close homologue is found in Genbank except that in *Streptomyces sp.* Mg1 with 66% identity.

**Table 1:** **Putative cytochrome P450s in *S. virginiae *IBL14 with their closest homologs**

**ID**^**a**^	**Size**^**b**^	**Best matches in the database**				
		**Species**	**Protein identifier**	**CYP family**	**AA overlap**^**d**^	**identity**^**e**^**%**
Svu001	464	*Streptomyces sp*. Mg1	ZP_05001937	new	598	66
		*Photobacterium profundum* 3TCK	ZP_01217946		113	25
Svu002	361	*Streptomyces virginiae*	ABR68806	105 L	134	100
		*Streptomyces clavuligerus* ATCC 27064	ZP_06769587		93.2	66
Svu003	439	*Streptomyces venezuelae* ATCC 10712	CCA59424	105C	608	77
		*Streptomyces cattleya* NRRL 8057	YP_004920090	105C	553	70
Svu004	398	*Streptomyces virginiae*	ABR68806	105 L	794	99
		*Streptomyces sp*. ACT50-5	BAG16627		529	69
Svu005	400	*Streptomyces sp*. C	ZP_07285089	105D	595	74
		*Streptomyces avermitilis* MA-4680	BAC75180	105D7	529	69
Svh01	399	*Streptomyces virginiae*	ABR68805	105C1	797	99
		*Streptomyces viridochromogenes* DSM 40736	ZP_07307444	105	703	87
Svu006	403	*Streptomyces virginiae*	ABR68807	107 L14	713	99
		*Streptomyces sp*. C	ZP_07284721	107 L14	611	87
Svu007	351	*Streptomyces sp*. C	ZP_07290554	107E	609	87
		*Streptomyces violaceusniger* Tu 4113	YP_004815015		540	77
Svu008	406	*Streptomyces sp*. C	ZP_07285026	107 L14	604	77
		*Streptomyces sp*. Mg1	ZP_04997607	107 L14	584	76
Svu009	415	*Streptomyces sp*. C	ZP_07286517	107 L	727	85
		*Streptomyces sp*. Mg1	ZP_04999247	107 L	484	59
Svu010	396	*Streptomyces sp*. Mg1	ZP_04997607	107 L14	578	74
		*Streptomyces sp*. C	ZP_07285026	107 L14	556	72
Svu011	405	*Streptomyces sp*. C	ZP_07287693	107 L	728	91
		*Streptomyces clavuligerus* ATCC 27064	ZP_05005324	107 L	601	75
Svu012	430	*Streptomyces sp*. C	ZP_07287209	107 L	808	92
		*Streptomyces sp*. Mg1	ZP_05000207	107 L	780	91
Svu013	396	*Streptomyces sp*. C	ZP_07287353	107 L	578	75
		*Streptomyces hygroscopicus subsp*. jinggangensis 5008	AEY86095		383	54
Svu014	395	*Streptomyces sviceus* ATCC 29083	ZP_06921933	107 L	961	80
		*Streptomyces venezuelae* ATCC 10712	CCA53921	107 L	549	79
Svu015	406	*Streptomyces sp*. Mg1	ZP_05001939	107 L	667	80
		*Streptomyces scabiei* 87.22	YP_003488837	107 L	640	77
Svu016	406	*Amycolatopsis editerranei* U32	YP_003767608	107 M	482	63
		*Actinomadura hibisc*	BAA23153		387	55
Svu017	368	*Streptomyces avermitilis* MA-4680	NP_823237	197A1	436	64
		*Streptomyces scabiei* 87.22	YP_003487606		389	59
Svu018	393	*Streptomyces venezuelae* ATCC 10712	CCA55152	121A	509	67
		*Mycobacterium tuberculosis* 02_1987	ZP_06504929	121A	464	57
Svu019	421	*Streptomyces sp*. C	ZP_07287311	124B	782	94
		*Streptomyces pristinaespiralis* ATCC 25486	ZP_06909795	124B	566	70
Svu020	416	*Streptomyces sp*. C	ZP_07289557	147A	731	91
		*Streptomyces peucetius* ATCC 27952	CAE53704	147A	667	79
Svu021	421	*Streptomyces sp*. C	ZP_07290439	152A	515	71
		*Streptomyces sp*. SirexAA-E	YP_004806454	152A	429	58
Svu022	412	*Streptomyces sp*. Mg1	ZP_05002011	154H	742	91
		*Streptomyces sp*. SirexAA-E	YP_004804189	154H	666	83
Svu023	409	*Streptomyces sp*. C	ZP_07285064	157A	773	93
		*Streptomyces sp*. Mg1	ZP_05002010	157A	734	88
Svu024	450	*Streptomyces sp*. Mg1	ZP_05002596	157C	723	82
		*Streptomyces hygroscopicus* ATCC 53653	ZP_07300920	157C	574	64
Svu025	89	*Streptomyces tubercidicus*	AAT45286	185A1	85.9	47
		*Actinosynnema mirum* DSM 43827	YP_003102184	185A	84.7	51
Svu026	409	*Streptomyces violaceusniger* Tu 4113	YP_004813101	191A	313	44
		*Rhodococcus opacus* B4	YP_002781958		300	43
Svu027	398	*Streptomyces sp*. C	ZP_07286547	191A	756	92
		*Streptomyces sp*. Mg1	ZP_04998169	191A	733	89
Svu028	446	*Streptomyces sp*. Mg1	ZP_04997583	191A	699	88
		*Streptomyces sp*. C	ZP_07290135	191A	692	90
Svu029	476	*Singulisphaera acidiphila* DSM 18658	ZP_09568426	197A	199	33
		*Streptomyces roseosporus* NRRL 11379	ZP_04712663	197A	191	32
Svu030	447	*Streptomyces sp*. C	ZP_07289871	197B	713	82
		*Streptomyces sp*. Mg1	ZP_05001362	197B	680	77
Svu031	710	*Streptomyces sp*. C	ZP_07284739	197B	353	79
		*Streptomyces clavuligerus* ATCC 27064	ZP_05006237		350	55
Svu032	416	*Streptomyces flavogriseus* ATCC 33331	YP_004921083	247A	693	81
		*Frankia alni* ACN14a	YP_712777	247A	573	70

^a^ The name of the putative CYPs in *S. virginiae* IBL14.

^b^ Amino acid number of putative CYPs.

^c^ Closest homologs in Genbank and the family classification of CYPs searched in CYPED.

^d^ Number of amino acid overlap, which exceeds the protein size, is due to the introduction of gaps during BLAST comparison.

^e^ The highest percent identity for a set of aligned segments to the same subject sequence.

### Features of CYPs from *S. virginiae* IBL14

Table 2 displays the three characteristic motifs of CYPs of *S. virginiae* IBL14. The critical residues are highlighted with bold fonts, which are threonine (T) in GXXTT motif of I-helix, glutamic acid (E) and arginine (R) in EXXR motif of K-helix and cysteine (C) in the GXXXCXG heme-binding domain signature, respectively.

**Table 2:** **A comparison of the conserved domain of putative CYPs in *S. virginiae *IBL14 with those of the same (sub)family in CYPED using ClustalW**

**ID**	**I-helix**	**K-helix**	**Heme binding motif**	**Accession numbers**
Svu001	Unidentified	**E**^335^TL**R**^338^	F^403^LPFGAGPRH**C**VG^415^	JX119062
Svu002	Unidentified	Unidentified	L^297^RVGVDRRL**C**CG^308^	
Svu003	G^276^LD**T**T^280^	**E**^314^LL**R**^317^	H^375^LGFGHGIHQ**C**LG^387^	JX119063
Svu004	G^237^HE**T**T^241^	**E**^275^SL**R**^278^	H^337^LGFGHGIHQ**C**LG^349^	JX119064
Svu005	G^247^HE**T**T^251^	**E**^285^LM**R**^288^	H^346^LAFGFGIHQ**C**LG^358^	JX119065
Svh01	G^235^FD**T**T^239^	**E**^273^LL**R**^276^	H^334^LAFSHGIHQ**C**LG^346^	EF646279
Svu006	G^277^HE**T**T^281^	**E**^315^ML**R**^318^	H^377^IAFGHGLHY**C**LG^389^	JX119066
Svu007	G^238^HE**T**T^342^	**E**^276^LL**R**^279^	H^339^LGFGHGVHH**C**LG^351^	JX119067
Svu008	G^236^HE**T**T^240^	**E**^275^ML**R**^278^	H^337^LAFGHGLHF**C**IG^349^	JX119068
Svu009	G^236^HK**T**T^240^	**E**^274^MQ**R**^277^	H^338^LGFGYGAHY**C**LG^350^	JX119069
Svu010	G^234^HE**T**T^238^	**E**^273^ML**R**^276^	H^335^LAFGHGIHF**C**IG^347^	JX119070
Svu011	G^242^HEAT^246^	**E**^285^LM**R**^288^	H^346^LTFGAGIHY**C**LG^358^	JX119071
Svu012	G^259^FE**T**T^263^	**E**^302^LL**R**^305^	H^364^LGYGHGIHY**C**LG^376^	JX119072
Svu013	G^237^SE**T**V^241^	**E**^275^LF**R**^278^	H^337^LALGHGVHY**C**LG^349^	JX119073
Svu014	G^234^HE**T**T^238^	**E**^272^LL**R**^275^	H^334^LAFGHGVHR**C**LG^346^	JX119074
Svu015	F^247^AP**T**T^251^	**E**^285^VV**R**^288^	Q^347^LSFGIGVHS**C**LG^359^	JX119075
Svu016	G^244^YH**T**T^248^	**E**^282^AL**R**^285^	H^345^LAFGAGIHF**C**LG^357^	JX119076
Svu017	G^207^FL**T**T^211^	**E**^245^GL**R**^248^	H^307^VAFGYGPHA**C**PG^319^	JX119077
Svu018	G^231^VIST^235^	**E**^269^LL**R**^272^	H^332^FSFGGGSHY**C**PA^344^	JX119078
Svu019	G^256^VE**T**T^260^	**E**^295^MI**R**^298^	H^356^LGFGGGGPHF**C**LG^369^	JX119079
Svu020	G^251^HE**T**T^255^	**E**^289^LL**R**^292^	H^351^LGLGSGIHS**C**FG^363^	JX119080
Svu021	T^247^WF**T**T^251^	**E**^281^VR**R**^284^	E^347^LIAQGGGNARTGHR**C**PG^364^	JX119081
Svu022	G^251^HE**T**T^255^	**E**^286^TL**R**^289^	H^349^ISFGHGPHV**C**PG^361^	JX119082
Svu023	G^238^HQP**T**^242^	**E**^276^VLW^279^	F^337^SFGHGEHRCPFPA^350^	JX119083
Svu024	A^247^FE**T**T^251^	**E**^284^QILW^288^	S^344^HLAFSSGPHE**C**PG^357^	JX119084
Svu025	Unidentified	Unidentified	H^50^LALGIGPHV**C**MG^62^	JX119085
Svu026	G^249^NE**T**T^253^	**E**^287^VL**R**^290^	H^348^LALGSGPHY**C**LG^360^	JX119086
Svu027	G^238^NE**T**T^242^	**E**^274^IV**R**^277^	H^335^LGFGGGGPHF**C**LG^348^	JX119087
Svu028	G^284^ND**T**V^288^	**E**^322^LL**R**^325^	H^383^VSFGDGPHV**C**LG^395^	JX119088
Svu029	A^242^HE**T**T^246^	**E**^297^TL**R**^300^	A^367^FMPFGGGPRT**C**LG^380^	JX119089
Svu030	G^259^HE**T**T^263^	**E**^314^AM**R**^317^	A^383^WFPFGGGPRA**C**IG^396^	JX119090
Svu031	G^499^HE**T**T^503^	**E**^545^TL**R**^548^	A^614^YLPFGIGPGPAWARSSRCGS^634^	
Svu032	A^252^NV**T**T^256^	**E**^290^GL**R**^293^	R^351^HGAFGFGPHF**C**IG^364^	JX119091

From the Table 2, we can find the I-helix is absent in *Svu*001(new family), and the I-helix and K-helix missing in *Svu*002 (105 L, often for hydroxylation activity) [29], which reflects I-helix is not absolutely essential for the CYP structure. The 2 members of CYP157 family *Svu*023 (E^276^VLW^279^)/157A and *Svu*024 (E^284^QILW^288^)/157C do not have arginine residue in K-helix like the CYP157C1 from *S. coelicolor* A3(2) having a motif E ^297^ QSLW [30] and the CYP157A2 and CYP157C2 from *S. avermitilis* exhibiting a ^257^EVLW motif and a ^257^EQSLW motif [26]. The CYP157 family proteins that lack consensus EXXR motifs but genetically are linked to their upstream conservons imply that they have functions linked to the upstream pathway(s) [30]. Besides, *Svu002*, *Svu018*, *Svu021*, *Svu023* and *Svu031* do not strictly follow the GXXXCXG motif of heme-binding.

### Multiple alignments and phylogenetic analysis

**Figure 1:**
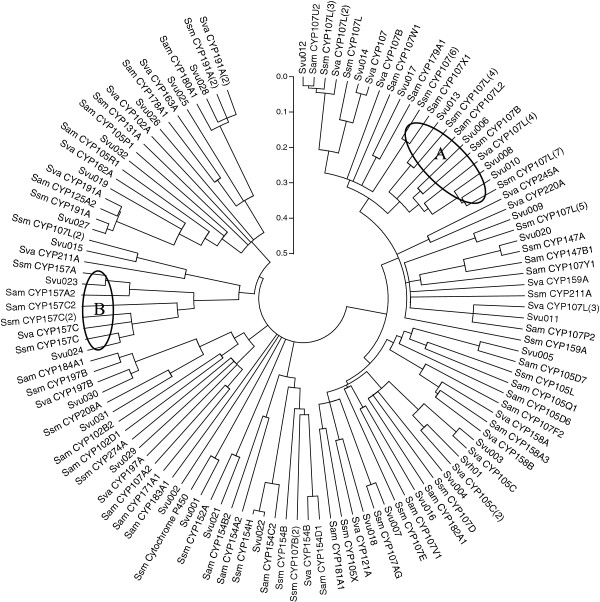
**Phylogenetic tree of the CYPs from *S. virginiae *IBL14 and three related bacteria.** Sequences were aligned using Clustal W and the tree was calculated and constructed using MEGA 5.0. (*Streptomyces sp*. Mg1, Ssm; *S. avermitilis* MA-4680, Sam; *S. venezuelae* ATCC 10712, Sva).

The phylogenetic tree of the combined CYPs of *S. virginiae* IBL14, *S. avermitilis* MA-4680, *S. venezuelae* ATCC 10712 and *Streptomyces sp.* Mg1 is presented in Figure 1. From Figure 1, we can find almost of all the CYPs in *S. virginiae* IBL14 are closely related to their homologues. More than 10 of CYPs from *S. virginiae* IBL14 are close to those from *Streptomyces sp.* Mg1 and the member (*Svu*001) of new CYP family found in *S. virginiae* IBL14 is only close to *Streptomyces sp.* Mg1. These results indicate that the CYPs from *S. virginiae* IBL14 are closer to those from *Streptomyces* sp. Mg1 than those from other *Streptomyces* spp, including *S. avermitilis* MA-4680 and *S. venezuelae* ATCC 10712. For the four species of *S. virginiae* IBL14, *sp*. Mg1, *avermitilis* MA-4680 and *S. venezuelae* ATCC 10712, the families CYP 107 and CYP157 (labeled with circle A and B in Figure 1, respectively) have more closely evolutionary relationship.

**Figure 2:**
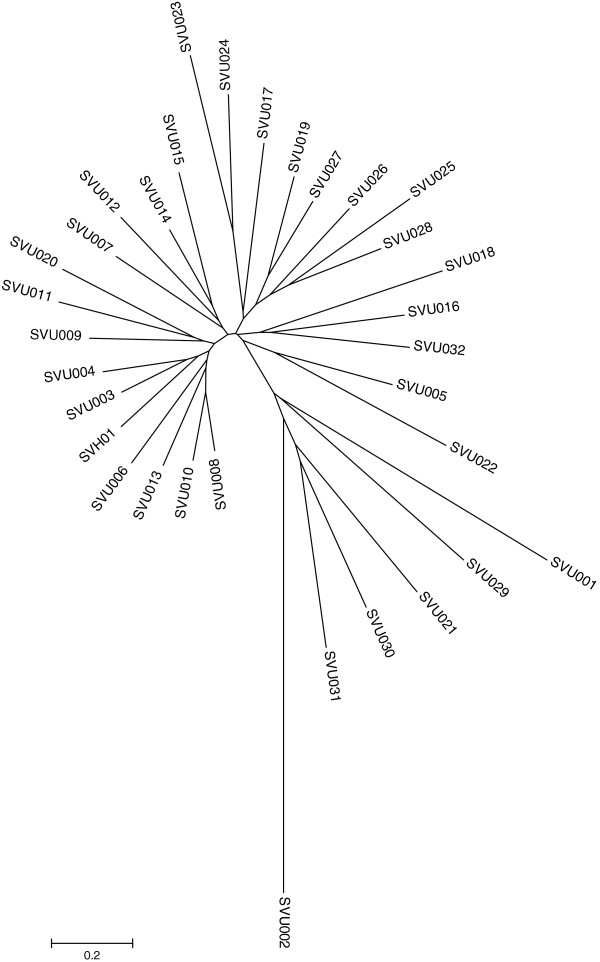
**A paralogous tree of all CYP sequences from *S. virginae *IBL14.**

Further, the paralogous relationship of the 33 CYPs in *S. virginiae* IBL14 was generated with the neighbor-joining methods (Clustal W and MEGA 5.0). From Figure 2, we can find that *svh*01 and *svu*03 and *svu*04 as well as *svu*022 and *svu*005 in *S. virginiae IBL14* have the closest homologous evolutionary relationship, respectively. It’s worth noting that most members belonging to the same CYP family are clustered together as expected, e.g., the 11 members of CYP107 family.

### The prediction of functions of CYPs in *S. virginiae* IBL14

A high identity over 70% among different protein sequences reasonably suggests that they may hold similar function [26]. As shown in the Table 1, we can find a sum of 26 CYP sequences of *S. virginiae* IBL14 have best matches to those of other *Streptomyces*, which are helpful in function prediction.

CYP105 and CYP107 are the most studied bacterial cytochromes which are associated with the degradation and biotransformation of a diverse array of xenobiotics and antibiotic biosynthesis. Analysis of CYPs sequence of *S. virginiae* IBL14 shows that there are 11 CYPs belonging to CYP107, five to CYP105, four to CYP197, three to CYP191, two to CYP157 and one to each other family, which indicates the diversity and importance of the two groups CYP105 and CYP107. The predicted functions of several putative CYPs in *S. virginiae* IBL14, combined with reported experimental evidences, were listed in Table 3.

**Table 3:** **Prediction of functions of several putative CYPs in *S. virginiae *IBL14**

**ID**	**Functions**	**Reference**
Svu003	Hydroxylation & O-demethylation	[15]
Svu005	N-demethylation & Hydroxylation	[31]
Svh01	Hydroxylation	[32] and this study
Svu006	Hydroxylation	[17]
Svu007	Hydroxylation	[16]
Svu019	Hydroxylation	[19]
Svu021	Hydroxylation& Decarboxylation	[33]
Svu022	Hydroxylation& O-dealkylation	This study

### CYPs in *S. virginiae* IBL14 and their ferredoxin reductase and ferredoxin

The catalytic activity of CYPs depends greatly on individual ferredoxin or/and ferredoxin reductase associated with. It was reported that there are three, six and four ferredoxin reductase genes and six, nine and two ferredoxin genes in *S. coelicolor* A3 (2), *S. avermitilis* and *S. peucetius*, respectively. In *S. coelicolor* A3 (2) only CYP105D5 is arranged in an operon with a ferredoxin gene [24]. In *S. peucetius* CYP147F is clustered with ferredoxin reductase [27]. In *S. avermitilis* both CYP105P1 and CYP105D6 are clustered with ferredoxin, CYP147B1 is arranged in an operon with a ferredoxin and ferredoxin reductase, CYP105Q1 is associated in an operon containing both a ferredoxin and ferredoxin reductase, and CYP102 is fused to a P450 reductase [26].

Three ferredoxin reductase genes and seven ferredoxin genes are found in *S. virginiae* IBL14 after annotation of *S. virginiae* IBL14 genome. That is, the activities of many of the CYPs in *S. virginiae* IBL14 are supported by different combinations with the three ferredoxin reductases and seven ferredoxins. Also in *S. virginiae* IBL14, *svu*005 (CYP105D), *svh*01 (CYP105C) and *svu*019 (CYP124B) is found to cluster with ferredoxin *svf*03, *svf*09 and *svf*07, respectively and *svu*020 (CYP147A) clustered with ferredoxin reductase *svfr*03 and ferredoxins *svf*06. The facts suggest that the functional realization of CYPs Svu005, Svh01, Svu019 and Svu020 needs the participation of electron transfer. The result of homology analysis by Blast-searching the Genbank are listed in the Table 4.

**Table 4:** **Putative ferredoxin reductases and ferredoxins in *S. viginiae *IBL14 with their closest homologs**

**ID**^**a**^	**Accession numbers**	**NO. nucleic acids**	**Match in the databases**^**b**^		
			**Species**	**Accession**	**Identity%**
Putative ferredoxin reductases					
*svfr*01	JX119052	453	*Streptomyces sp.* C	ZP_07290734	94
			*Streptomyces pristinaespiralis* ATCC 25486	ZP_06911868	92
*svfr*02	JX119053	463	*Streptomyces sp.* C	ZP_07285271	87
			*Streptomyces sp.* Mg1	ZP_05002250	84
*svfr*03	JX119054	464	*Streptomyces sp*. C	ZP_07289558	94
			*Streptomyces peucetius* ATCC 27952	CAF33360	84
Putative ferredoxins					
*svf*03	JX119055	219	*Streptomyces sp*. C	ZP_07285090	84
			*Streptomyces viridochromogenes* DSM 40736	ZP_07308348	79
*svf*04	JX119056	1143	*Streptomyces sp.* Mg1	ZP_04996989	83
			*Streptomyces griseoflavus* Tu4000	ZP_07315146	83
*svf*05	JX119057	234	*Streptomyces sp.* C	ZP_07286537	88
			*Streptomyces sp.* Mg1	ZP_05002165	79
*svf*06	JX119058	231	*Streptomyces peucetius* ATCC 27952	ACE73829	62
			*Streptomyces hygroscopicus subsp*	AEY87986	61
*svf*07	JX119059	600	*Streptomyces sp.* C	ZP_07287304	98
			*Streptomyces venezuelae* ATCC 10712	CCA56325	94
*svf*08	JX119060	315	*Streptomyces sp.* C	ZP_07285869	89
			*Streptomyces peucetius* ATCC 27952	ACE73824	88
*svf*09	JX119061	243	*Streptomyces cattleya* NRRL 8057	YP_004920089	63
			*Streptomyces diastaticus*	AAR16520	61

^a^ The name of gene in *S. virginiae* IBL14.

^b^ Homologues searched in Genbank.

### Regulatory elements and functional genes clustered with CYPs

The CYPs in *S. peucetius* ATCC27952 clustered with regulatory elements were reported [27]. In the annotations of gene arrangement around the putative CYPs on the *S. virginiae* IBL14 chromosome, *svu*022, *svu*023 and *svu*024 were found to cluster with the genes of ATP/GTP binding proteins (having a phosphate-binding loop for energy requiring metabolic reactions) [34], *svu*001, *svu*015 to cluster with LysR-family transcriptional regulator (regulating a diverse set of genes, including those involved in virulence, metabolism, quorum sensing and motility) [35], *svu*011 to cluster with two component transcriptional regulators and LuxR family (quorum sensing signals in Gram-negative bacteria often regulated by acylated homoserine lactones) [36], *svu*018 to cluster with a transcriptional regulator, AraC family (transcriptional regulators having diverse functions ranging from carbon metabolism to stress responses to virulence) [37] and two component transcriptional regulators, LuxR family and *svu*020 to cluster with the ATP-binding protein *fbpC* and TetR-family transcriptional regulators (among bacteria with an HTH DNA-binding motif for the transcriptional control of multidrug efflux pumps, pathways for the biosynthesis of antibiotics, response to osmotic stress and toxic chemicals, control of catabolic pathways, differentiation processes, and pathogenicity) [38].

As described above, the CYPs in *S. virginiae* IBL14 chromosome are responsible for the transcriptional regulation of many functional genes related with primary, secondary metabolism, as well as the responses to environmental factors as expected. Besides, CYPs are clustered with other functional genes. *svh*01 is adjacent to the genes of MdlB, ABC-type multidrug transport system, ATPase and permease components, which may be involved in the transportation of substrates [39]. *svu*009 lies next to alcohol dehydrogenase, suggesting that *svu*009 may take part in alcohol bioconversion and biodegradation. *svu*013 is next to 4, 5-DOPA dioxygenase which is a member of the class III extradiol dioxygenase family (a group of enzymes which use a non-heme Fe (II) to cleave aromatic rings between a hydroxylated carbon and an adjacent non-hydroxylated carbon), suggesting that the combination of *svu*013 and 4, 5-DOPA dioxygenase may be responsible in biodegradation of substrates with aromatic rings. *svu*026 is adjacent to MbtH-like protein which is found in known antibiotic synthesis gene clusters [40]. The cholesterol oxidase ChoL from *S. virginiae* IBL14 in the bioconversion and biodegradation of diosgenin responsible for the conversion of diosgenin to diosgenone (a 4-ene-3-keto steroid) via a couple of C3-dehydrogenation and C4-5-isomerization was reported [41]. In *S. virginiae* IBL14 the gene encoding Svu004 (CYP105L) clusters with the genes of putative ferredoxin Svfr2 and cholesterol oxidase (ChoL), suggesting that the cytochrome P450 joins with the cholesterol oxidase ChoL to catalyze the oxidation of cholesterol and its structural analogs. In conclusion, CYPs from *S. virginiae* IBL14 may have multiple functions in secondary metabolism, including hydroxylation, dehydrogenation, ring-cleavage, transportation, etc.

### Functional identification and characteristics of *svh*01 and *svu*022

To elucidate all putative CYPs’ functions in *S. virginiae* IBL14, four CYP genes of the strain IBL14 were firstly selected. Among them, the functional identities of CYP genes *Svh01*(105C1) and *svu*022 (154H) has been finished.

The cytochrome P450 Svh01 (responsible for the C25-hydroxylation of diosgenin) [32] belongs to the class I (prokaryotic/mitochondrial) P450 system based on a taxonomic split, in which electrons are transferred from NADPH or NADH to ferredoxin reductase and ferredoxin. Sequence analysis revealed the complete sequence of *svh*01 with ATG as the start codon has 70% G + C content. The sequence of possible ribosome-binding site is located on the upstream of *svf*09 (a coenzyme of Svh01).

**Figure 3:**
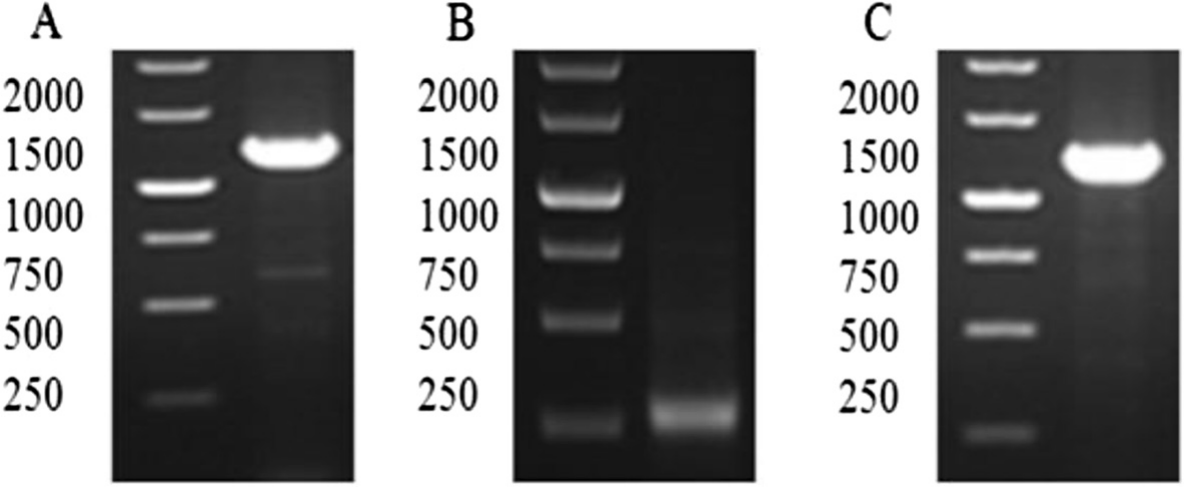
**DNA analysis of *svh*01, *svf*09 and *svu*022 cloned from *S. virginiae *IBL14. A**, **B** and **C** are the PCR results of *svh*01, *svf*09 and *svu*022, respectively.

Both *svh*01 and *svf*09 contain 1200 bp and 243 bp, respectively, based on sequence analysis. To obtain the expressed products of them, both *svh*01 and *svf*09 sequences were first ligated into a pET22b vector in a cluster to generate the expression plasmid pET22b-*svh*01-*svf*09 that was then cloned into *E. coli* JM109 (DE3) to form a recombinant strain *E. coli* IBL161 [JM109 (DE3)/pET22b-*svh*01-*svf*09]. The PCR results of *svh*01 and *svf*09 from the recombinant strain *E. coli* IBL161 were analyzed by gel electrophoresis (Figure 3A and B) and also confirmed by gene sequencing.

The *svu*022 with a G + C content of 73% (clustering with the gene of ATP/GTP binding protein) consists of 1239 nucleotides. Similarly, the complete sequence of *svu*022 was first inserted to the shuttle plasmid pHCMC05 to form the recombinant plasmid pHCMC05-*svu*022, and then cloned in *B. subtilis* WB800N (improving the extracellular expression level of Svu022 for the analysis of enzymatic biotransformation) to produce the recombinant strain *B. subtilis* IBL 241 [WB800N/pHCMC05-*svu*022]. The PCR result of *svu*022 from the recombinant strain *B. subtilis* IBL 241 is shown in Figure 3C.

**Figure 4:**
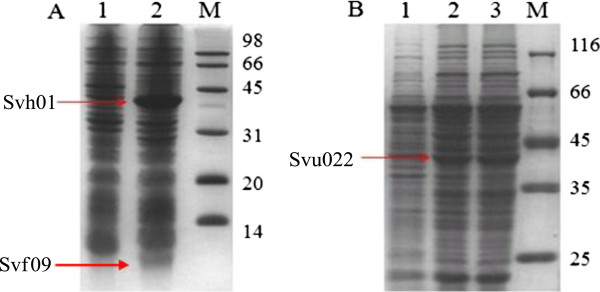
**SDS-PAGE analysis of Svh01 and Svu022.** (**A**) Lane 1, sample from JM109 (DE3)/pET22b cells; Lane 2, sample from *E. coli* IBL161. (**B**) Lane 1, WB800N/pHCMC05 cells; Lane 2 and 3, *B. subtilis* IBL 241.

Svh01 (105C1) is a peptide of 399 amino acids, with a molecular weight of 44.04 kDa and a pI value of 4.97 estimated by the ExPASy (a computing pI/MW tool). To obtain its expressed product and study product characteristics, the recombinant strain *E. coli* IBL161 was incubated and induced. The expression of Svh01 was shown in Figure 4A. From the SDS-PAGE, we can find that the two distinctly additional protein bands should be Svh01 with an about MW of 44 kDa and Svf09 with an about MW of 8.0 kDa, respectively. The further functional identification of the Svh01/FcpC of *S. virginiae* IBL14, hydroxylating the C25-tertiary carbon of diosgenin to form isonuatigenone, was experimentally confirmed [32].

**Figure 5:**
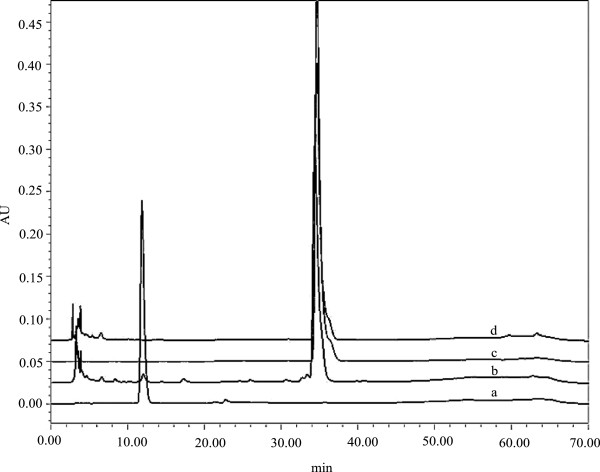
**The profiles of the transformation of β-estradiol by strain *B. subtilis *IBL 241 in HPLC. a**: standard estriol; **b**: sample from *B. subtilis* IBL 241; **c**: standard β-estradiol; **d**: sample from *B. subtilis* WB800N.

Svu022 (154H) is a deduced protein of 412 amino acids which shares 91% identity with that in *Streptomyces sp*. Mg1. The estimation of MW and pI of SVU022 are 44.59 kDa and 5.00, respectively. Similarly, the recombinant strain *B. subtilis* IBL 241 was incubated and induced to study the product expression and its characteristics. The expressed result of Svu022 from the recombinant strain *B. subtilis* IBL 241 was shown in Figure 4B. The SDS-PAGE displays a distinct protein band with about MW of 45.0 kDa as expected. The further experimental results from TLC, HPLC and LC/MS indicated that the CYP Svu022 enables to biotransform β-estradiol into estriol. Figure 5 shows the profiles of the biotransformation of β-estradiol by strains *B. subtilis* WB800N and *B. subtilis* IBL 241 in HPLC. The functional identification of the Svu005 (CYP105D) and Svu019 (CYP124B) is in progress.

## Conclusion

*S. virginiae* IBL14 contains 33 putative CYPs, 7 ferredoxins and 3 ferredoxin reductases in its 8.0 Mb linear chromosome. Most of the CYPs in *S. virginiae* IBL14 belong to the CYP107 (11 members) family and CYP105 (5 members) family. Compared phylogenetically with CYPs from 3 typical *Streptomycete* spp., *S. virginiae* IBL14 appears to be closest to those of *Streptomyces* sp. Mg1.

Further analysis showed that among all of the 33 CYPs in *S. virginiae* IBL14, three CYPs are clustered with ferredoxins, one with ferredoxin and ferredoxin reductase and three CYPs with ATP/GTP binding proteins, four CYPs arranged with transcriptional regulatory genes and one CYP locates on the upper of ATP-binding protein and transcriptional regulators as well as four CYPs associated with other functional genes involved in secondary metabolism and degradation.

The new characteristics found in CYPs from *S. virginiae* IBL14 suggest that the EXXR motif in the K-helix is not absolutely conserved in CYP157 family as reported [30] and I-helix not absolutely essential for the CYP structure. Particularly, one new family was found based on the CYP *svu*001 in *S. virginiae* IBL14 which shares 66% identity only to that from *Streptomyces* sp. Mg1.

Two recombinant strains *E. coli* IBL161 [JM109 (DE3)/pET22b-*svh*01-*svf*09] and *B. subtilis* IBL 241 [WB800N/pHCMC05-*svu*022] were constructed and subsequently their functions were identified, respectively. Experimental results showed that both CYP Svh01 and CYP Svu022 are two hydroxylases, capable of bioconverting diosgenone into isonuatigenone and β-estradiol into estriol, respectively.

## Methods

### Strains and plasmids

*S. virginiae* IBL-14 (CCTCCM 206045) [42] as the strain of interest was used for the Cytochrome P450 gene identification and functional analysis. *E. coli* JM109, JM109 (DE3) and *B. subtilis* WB800N were used as the host for plasmid construction and target protein expression in the functional identification of the CYPs, respectively. The vector pET22b was used for cloning and expression of genes of interest in *E. coli*. The shuttle plasmid pHCMC05 was used for the expression of target proteins in *B. subtilis* (a GRAS strain by FDA). The features of the bacterial strains and plasmids used in this study are listed in Table 5.

**Table 5:** Microorganisms and plasmids used in this study

**Strains**	**Relevant properties**	**Source**
*Escherichia coli*		
JM109	Cloning host, genotype:endA1, *rec*A1, *gyr*A96, *thi*, *hsd*R17 (rk^–^, mk^+^), *rel*A1, *sup*E44, (*lac-proAB*), [F’ *tra*D36, *pro*AB, *laq*I^q^ZΔM15]	Promega
JM109 (DE3)	Expression host, genotype:*end*A1, *rec*A1, *gyr*A96, *thi*, *hsd*R17 (rk^–^, mk^+^), *rel*A1, *sup*E44, λ–, Δ(*lac-proAB*), [F’, *tra*D36, *pro*AB, *lac*I^q^ZΔM15], lDE3	Promega
*Bacillus subtilis*		
WB800N	Secretion host with resistance to neomycin, genotype: *npr*E *apr*E *epr bpr mpr* :: *ble npr*B :: *bsr vpr wpr*A :: *hyg cm* :: *neo*; NeoR	Mo Bi Tec
*Streptomyces virginiae*		
IBL14	Wild type	Our lab
Plasmids		
pET22b	Expression vector in *E. coli*	Novagen
pHCMC05	Shuttle plasmid	BGSC
pET22b-*svh*01-*svf*09	The fragment of *svh*01 and *svf*09 were digested with *Nde*I/*EcoR*Iand *EcoR*I/*Hind* Ш, respectively, and ligated into the *Nde*Iand *Hind* Ш sites of pET22b	This study
pHCMC05-*svu*022	The gene of *svu*022 digested with *BamH*I/*Sma*Iligated into *BamH*I/*Sma*Idigested pHCMC05	This study

### Media and cultivation

Luria-Bertani (LB) medium was used for plasmid construction and protein expression. A final concentration of 70 μg/ml ampicillin was supplemented into the medium when *E. coli* IBL161 [JM109 (DE3)/pET22b-*svh*01-*svf*09] and *E. coli* IBL152 [JM109/pHCMC05-*svu*022] were cultivated. A final concentrations of 25 μg/ml chloramphenicol was added to the medium when *B. subtilis* IBL 241 [WB800N/pHCMC05-*svu*022] was cultivated. The cultivating procedure of *S. virginiae* IBL-14 has been described previously [42]. Diosgenin in 95% purity (J&K Chemical Ltd, China) and β-estradiol in 98% purity (J&K Chemical Ltd, China) were dissolved in anhydrous ethanol before adding into medium.

### Sequencing and *in-silico* identification analyses of CYPs

The *S. virginiae* IBL14 genome sequencing was performed at 454 platform (Encode Genomics Co. Ltd., Suzhou, China) for the first time (sequence data will be published step by step). All of the ORFs of this genome were predicted using glimmer3.0 and prodigal, respectively. To dig out all possible CYP gene function information in *S. virginiae* IBL14, the genome sequence of the strain was compared with the SWISSPROT, TrEMBL, KEGG databases by using Blastp and the CDD and COG databases by using Rpsblast, respectively.

The deduced amino acid sequences of the putative CYPs of *S. virginiae* IBL14 were aligned with the CYPs from *S. avermitilis* MA-4680, *S. venezuelae* ATCC 10712 and *Streptomyces sp.* Mg1 by using ClustalW [43]. Then the molecular evolution and phylogenetic analyses by neighbor-joining methods were carried out using MEGA5.0 [44]. To forecast the possible functions involved in secondary metabolism, comparison between all putative CYPs of *S. virginiae* IBL14 with those in other organisms based on homologues was done by using Blastp too.

Using the three motifs as described above as criteria, the CYP gene candidates of *S. virginiae* IBL14 were blast searched against GenBank non-redundant protein database to identify their closest bacterial homologues and tentatively distribute all of the CYPs of *S. virginiae* IBL14 into the corresponding family or subfamily [26]. Similar procedure was performed to the putative ferredoxin and ferredoxin reductase genes to identify their closest bacterial homologues.

### Construction and cloning of expression plasmids

The genes of *svh*01, *svf*09 and *svu*022 from the genomic DNA of *S. virginiae* IBL14 were amplified by using PCR method (Pfu DNA Polymerase, Fermentas, Thermo Fisher Scientific Inc.) and the primers used are listed in Table 6. The PCR products of *svh*01 and *svf*09 were digested with *Nde*I/*EcoR*Iand *EcoR*I/*Hind* III, respectively, then ligated into a pET22b vector, and finally transformed to the host bacterium *E. coli* JM109 (DE3). Similarly, the PCR product of *svu*022 was digested with *BamH*I/*Sma* I, then ligated into a shuttle plasmid pHCMC05 and finally transformed to *B. subtilis* WB800N.

**Table 6:** The PCR primers used in this study

**Primer**	**Primer sequence**^**a **^**(5**^**′ **^**to3**^**′**^**)**	**Restriction site**
pSVH01F	GCCCCCCATATGAGTGAGTCCCTCCACACCGTC	*Nde*I
pSVH01R	GGAGGAATTCACTTCGCGTCCCAGGTG	*EcoR*I
pSVF09F	CCGGAATTCGGGACGCGAAGTGAGCGCGG	*EcoR*I
pSVF09R	CCCAAGCTTTCAGGCGGAGGGTGGGCGG	*Hind* III
pSVU022F	CTGGATCCATGAGCTGCCCGATCGACC	*BamH*I
pSVU022R	CCTAAGCTTTCAGGGGTGCAGGCGTACCG	*Sma*I

^a^The underlined sequence are recognition sites of restriction enzymes and the nucleotides before it are the protected bases. All primers are designed by Primer Premier 5.0 and verified by Oligo 7.0.

### Expression and analysis of target proteins

0.3 ml (inoculation ratio of 1%) of the overnight culture of *E. coli* IBL161 as seed was inoculated in 30 ml LB medium (containing 70 μg/ml ampicillin) and then cultivated at a shaking speed of 200 rpm at 37°C. The expression of target protein was induced by adding 0.2 mM IPTG when the OD value reached 0.5 ~ 0.6 at 600 nm. Then the culture was continuously cultivated for another 24 h at 25°C at a speed of 200 rpm in a rotary shaker. Similarly, the overnight culture of *B. subtilis* IBL 241 was inoculated with 1% ratio in 30 ml LB medium (25 μg/ml chloramphenicol, 200 rpm at 30°C). After adding 0.2 mM IPTG in logarithmic growth phase, the culture was continuously cultivated for another 48 h at the same conditions. The harvested recombinant cells were resuspended and subjected to ultrasonication in 50 mM PBS (pH 7.4), and then centrifuged at 6000 rpm for 5 min. The supernatant was analysed by SDS-PAGE.

### Biotransformation and product extraction

One milliliter of β-estradiol/diosgenin (a final concentration of 0.2 mg/ml) for each flask was added for biotransformation analysis after *E. coli* IBL161 was induced by IPTG at 25°C for 2 h. After cultivated for another 24 h under the same conditions, the cultures were extracted two times with a half volume of 100% ethyl acetate (Sinopharm Chemical Reagent Co., Ltd). The extracts were evaporated to dryness, then re-dissolved in 1 ml anhydrous ethanol, and finally detected and analyzed (thin layer chromatography/TLC, high performance liquid chromatography/HPLC and liquid chromatography–mass spectrometry LC-MS).

### DNA and protein analytical methods

DNA electrophoresis for recombinant plasmid analysis was carried out in agarose gels at 110 V for 30 min [45]. SDS-PAGE with a 15% (w/v) acrylamide gel for expressed protein analysis was run at 110 V for 2 h according to Schagger’s publication [46]. The bands were visualized by Coomassie R-250 staining.

### HPLC analysis of biotransformation products

To identify and analyze the metabolites, high performance liquid chromatography (HPLC) was carried out. Simply, the sample of 10 μl was first loaded onto 250 mm Symmetry C 18 (4.6 mm × 250 mm, Waters Co., USA) and eluted with ethanol/water (60/40, v/v). The flow rate, the wavelength for UV-detection and the temperature of the column on the HPLC system (Breeze 1525 series, Waters Co., USA) were set at 1 ml/min, 245 nm and 35°C, respectively. The products after biotransformation were qualitatively and quantitatively analyzed by comparing with corresponding standard material.

## Competing interests

The authors declare that they have no competing interests.

## Authors’ contributions

Z-ZL carried out the bioinformatic and genomic analyses and experiments and drafted the manuscript. X-FL carried out the experiments and bioinformatic and genomic analyses, WY, XD, JY, S-LZ, ML, LX participated in the experiments or interpretation of results. W-YT contributed to study design, data analysis and writing of the manuscript. All authors read and approved the final manuscript.
